# The Effect of Three Different Disinfection Materials on Alginate Impression by Spray Method

**DOI:** 10.5402/2012/695151

**Published:** 2012-07-25

**Authors:** Hamid Badrian, Ehsan Ghasemi, Navid Khalighinejad, Nafiseh Hosseini

**Affiliations:** ^1^Dental Implant Research Center, School of Dentistry, Isfahan University of Medical sciences, 81746-73461 Isfahan, Iran; ^2^Department of Prosthodontic, Torabinezhad Center Research, Dental School, University of Medical Sciences, 81746-73461 Isfahan, Iran; ^3^Department of Microbiology, School of Medicine, Isfahan University of Medical Sciences, 81746-73461 Isfahan, Iran

## Abstract

*Introduction*. The aim of this study was to investigate the effect of three different types of disinfectant agents on alginate impression material after 5 and 10 minutes. *Method and Materials*. In this *in vitro* experimental study, 66 circular samples of alginate impression material were contaminated with *Staphylococcus aureus*, *Pseudomonas aeruginosa*, and *Candida albicans* fungus. Except for control samples, all of them were disinfected with sodium hypochlorite 0.525, Deconex, and Epimax by way of spraying. Afterwards, they were kept in plastic bags with humid rolled cotton for 5 and 10 minutes. The number of colonies was counted after 24 and 48 hours for bacteria and after 72 hours for fungus. Statistical Mann-Whitney test was used for data analysis (**α** = 0.05). *Results*. After 5 minutes, Epimax showed the highest disinfection action on *Staphylococcus aureus* as it completely eradicated the bacteria. The disinfection capacity of different agents can be increased as time elapses except for *Pseudomonas aeruginosa* which was eradicated completely in both 5 and 10 minutes. *Conclusion*. This study revealed that alginate can be effectively disinfected by three types of disinfecting agents by spraying method, although Epimax showed the highest disinfection action after 10 minutes compared to other agents.

## 1. Introduction

Dentists, dental materials, and dental laboratories are exposed to different types of pathogenic microorganisms. Impression materials, impression trays, and poured stone cast have been said to be the main source of cross infection between patients and dentists [1].

New researches have shown that 67% of materials sent to dental laboratories are infected by various microorganisms [2]. The most frequently identified microorganisms are *Streptococcus* species, *Staphylococcus* species, *Escherichia coli* species, *Actinomyces* species, *Antitratus* species, *Pseudomonas* species, *Enterobacter* species, *Klebsiella pneumonia,* and *Candida* species [3]. Taking this into account, we should make an effort to eliminate most of these microorganisms and reduce the rate of infection transmission in dental laboratories. The International Dental Federation (IDF) insists on disinfecting all impressions taken from patients before sending them to laboratories [4]. Also the American Dental Association (ADA) has advised all dental stuffs to disinfect patients' impression trays [5]. In some studies, it has been declared that washing the impression materials with tap water only removes 40% of bacteria; however, some studies reported that it has the capacity to reduce 90%, microorganisms [6]. The most common chemical disinfectants which are used by dentists are alcohols, aldehydes, chlorine combination, phenols, biguanides, iodide combinations, and ammonium [7]. There are two common methods to disinfect dental materials: (1) immersion and (2) spraying [6].

Disinfection by soaking in chemical materials has been shown to cover all surfaces of impression materials in one time [8], while spraying is not capable of disinfecting all surfaces effectively and also cannot cover all undercuts. Contrary to immersing, spraying can significantly reduce the amount of distortion [6]. Some impression materials, like alginate which is commonly used in dentistry [9], absorb water and distort when they are immersed in disinfectant materials [10] due to their hydrophilic properties.

In Westerholm et al. study in 1992 [11], the efficacy of eight different disinfectant agents was assessed; among them Sporicidin and 0.525% sodium hypochlorite effectively eradicated *Staphylococcus aurous* in all samples.

In another study, Rueggeberg et al. found that spraying disinfectants on alginate does not cause dimensional distortion in poured stone casts compared to casts from water-rinsed controls. It was shown that immersion method cause dimensional distortion in both anterior and posterior segments. Both spraying and immersing decrease surface details to the same extent. The antimicrobial effect of spraying and immersing methods was almost equal while mere water rinsing showed no significant disinfection effect [12].

Ghahramanloo et al. in 2009 investigated the antimicrobial effect of sodium hypochlorite 0.525%, Deconex, and Sanosil. They concluded that the use of 0.525% sodium hypochlorite spray on the surface of alginate effectively disinfect 96.6% of the samples [9].

Since none of the mentioned disinfection protocols have been accepted as a standard gold for disinfecting dental materials and the presence of hazardous microorganism on dental impression can impose detrimental effects, the present study was designed to investigate the disinfection effect of Deconex Solarsept solution, sodium hypochlorite 0.525%, and Epimax on alginate impression material in 5 minutes and 10 minutes.

## 2. Methods and Materials

The present randomized experimental study was carried out with the cooperation of Department of Microbiology the School of Dentistry, of Isfahan University of Medical Sciences aiming at evaluating the disinfection effect of: sodium hypochlorite %0.525 (Chloran, Tehran, Iran), Deconex (Borer chemie, Switzerland), and Epimax (Emad, Isfahan, Iran) on the alginate impression material (Zhermak, Rome, Italy).

### 2.1. Sampling Methods

An appropriate mixture of water and alginate powder was prepared in a bowl with a sterile spatula according to the manufacturers' instructions.

Then the mixture was poured into a 5 cc sterile syringe; after the required time for material setting, the impression material was cut off and removed with a number 10 surgical blade from the end part of the syringe to different slices with 2 mm thickness. Eventually, 66 alginate samples were prepared. In order to assure that the samples were kept sterile during preparation, three samples were selected as negative controls (blank) and were incubated on TSB culture for 24 to 48 hours, after which the bacterial growth was examined. To investigate the effect of different disinfectant materials, 21 samples were used for each bacterial species. From total 21 samples, sodium hypochlorite %0.525 was used to disinfect three of them for five minutes and three others for 10 minutes. Three samples were disinfected with Deconex for five minutes and three others for 10 minutes, and three samples were selected for disinfecting with Epimax for five minutes and three others for 10 minutes. At the last, three more samples were used as positive controls to check for any microbial pollution.

### 2.2. Preparation of Bacterial Suspension and Yeast

For each type of susceptibility testing, a standard inoculum of bacteria must be used.

The standard inoculums were prepared to match the turbidity of 1.5 × 10^8^ cfu/mL (equivalent to 0.5 McFarland) by transferring 1-2 colonies of 18–24 hours cultures to TSB medium and incubating at 35°C. For *Candida albicans* fungus, the samples were taken from 48 hour Saborosa and Dextrose agar cultures.

### 2.3. Contamination of Samples

To evaluate the disinfection effect of abovementioned three substances, samples were separately polluted with microbial solutions of *Staphylococcus aureus* (ATCC29213), *Pseudomonas aeruginosa* (ATCC27853), and *Candida albicans* fungus (PTCC5027). The impressions were put in sterile test tubes separately with 1 cc of microbial suspension each and then incubated at 35°C for one hour.

### 2.4. Disinfection of Samples and Microbiological Surveys

After contamination, all samples were rinsed with sterile distilled water for 30 seconds. In order to disinfect all samples except controls, either sodium hypochlorite %0.525,Deconex, or Epimax were used on each sample applying spray method for 10 puffs in 15 seconds. Then the samples were put into sterile plastic bags containing sterile humidified cotton to form a moisturized environment for 5 and 10 minutes.

Trypsin protease, which is able to isolate the microbes from contaminated environments, was used. The recommended time and concentration for the effective use of trypsin is 60 minutes and 2%, respectively. This time concentration is based on the maximum microorganisms which can be isolated from the samples. After washing the samples with sterile distilled water for 30 seconds, they were put in trypsin 2% solution for 60 minutes. The suspensions of 1/2 and 1/4 trypsin solution were then prepared. Using 100 micro liter sampler, these samples were transferred to Mueller-Hinton agar for the *Pseudomonas aeruginosa* and *Staphylococcus aurous*. Also Saborow Dextrose agar (SDA) medium was used to investigate the presence of *Candida albicans* fungus. Using a Pasteur pipet bent with heat at 90 degrees, the samples were spread on cultures. After 24 and 48 hours incubation, the grown bacterial colonies on culture were counted. The grown fungus colonies of *Candida albicans* on SDA were counted after 72 hours.

SPSS software was used for data analysis edition 11.5, and Mann-Whitney test was conducted to compare the efficacy of different disinfectant materials.

## 3. Results

According to Table 1, the disinfection action of three mentioned disinfectants showed no significant difference after 5 minutes for *Candida albicans* and *Pseudomonas aeruginosa*, however, this difference was significant for *Staphylococcus aureus*. (*P* value <0.05).

It was observed that Epimax is more efficient in eradicating *Staphylococcus aureus* compared to two others disinfectant agents. Also Deconex showed significantly higher disinfectant action in removing *Staphylococcus aureus* compared to 0.525% hypochlorite sodium. 

Table 2 illustrate the bacterial disinfection capability of the three above-mentioned disinfectants after 10 minutes. There was no significant difference in disinfection capability of the above disinfectants on *Candida albicans* and *Pseudomonas aeruginosa* and neither in disinfection ability of Deconex and Sodium Hypochlorite 0.525% on *Staphylococcus aureus*. For Deconex-Epimax and hypochlorite sodium-Epimax, there is a significant difference in eradicating *Staphylococcus aureus* (*P* < 0.05). Epimax also completely eradicated *Staphylococcus aureus* with higher rate compared to two other disinfectants. 

Table 3 displays bacterial disinfection ability of disinfectants in preventing microorganisms growth. Epimax could eradicate *Staphylococcus aureus* effectively (100%) after 5 minutes. %0.525 sodium hypochlorite and Deconex eliminated 97.12% and 95.39% of *Staphylococcus aureus*, respectively. Epimax completely prevented the growth of microorganisms in 10 minute (100%).

## 4. Discussion

Dentists practicing dentistry encounter potentially harmful microorganisms. Patients are the most important source of microorganisms [13]. Studies indicate that the surface of impressions taken out of the mouth is polluted with bacteria [14–17]. 

Egusa and colleagues in 2008 showed that alginate impressions taken from patients mouths contain hazardous microorganisms like *Staphylococcus aureus*, methicillin resistant *Staphylococcus*, *Candida albicans*, and *Pseudomonas aeruginosa* with rate of 55.6%, 25.9%, 25.9%, and 5.6%, respectively [18]. These are opportunistic pathogens that can be spread and transferred through the oral cavity [18]. *Candida* causes common opportunistic infections known as oral candidiasis found in patients with immune deficiency [19]. *Pseudomonas aeruginosa* is a deadly infectious agent that exists epidemically on hospital instrument [18]. However, studies show that among the population, the spreading rate of *Staphylococcus aureus* to the nasopharynx is only 10% [13]. This is the reason that in this survey *Staphylococcus aureus, Candida albicans*, and *Pseudomonas aeruginosa* were selected to investigate the disinfection efficacy of disinfectant agents. In the present study, all three kinds of disinfectant materials could efficiently eradicate all kinds of microorganisms, but Epimax showed more promising results compared to two other disinfectants. Although Epimax represented a significant difference with other disinfectants in 5 and 10 minutes in eradicating *Staphylococcus aureus* from alginate samples, there was no significant difference in its capacity in eradicating other microorganisms compared to other agents.

In the present survey, 0.525% hypochlorite sodium agent which is common in housework was used. This disinfectant agent could efficiently prevent microorganisms' growth and disinfect the impression materials. Westerholm [11] and Rueggeberg [12] and colleagues also showed that spraying sodium hypochlorite can effectively disinfect the impression materials. In Westerholm study, it was showed that Sodium hypochlorite can absolutely (99.99%) prevent the growth of *Staphylococcus aureus* [11], and these results are in agreement with the results of the present study as this material eradicated 97.12% and 98.84% of *Staphylococcus aureus* after 5 and 10 minutes, respectively. In another study by Ghahramanloo and colleagues, spraying sodium hypochlorite 0.525% could disinfect samples effectively (96.6%) in 10 minutes [9]. 

Deconex is an alcohol based disinfectant agent that in this survey could effectively disinfect impression materials. This agent exerts its effect mostly on *Pseudomonas aeruginosa* since it eradicated 99.27% and 100% of *Pseudomonas aeruginosa* after 5 and 10 minutes, respectively. In Hoseini et al. study [20], it was reported that Deconex is quite effective against *Pseudomonas aeruginosa* and *Staphylococcus aureus*. These results are also in accordance with the results of the present study. Contrary to our results in a study by Ghahramanloo et al., this agent could eradicate only 70.4% of microorganism [9]. It can be postulated that the use of a more resistant type of bacteria can explain this difference. Also it was shown that the disinfection capacity of Deconex can increase from 5 to 10 minutes. 

In this survey, for the first time the antimicrobial effect of Epimax on impression materials was investigated. Epimax is a broad spectrum hydrogen peroxide based products. The most important feature of Epimax is that it does not make bacteria resistant against disinfectant materials and it is highly biocompatible and does not induce any allergic reactions. Interestingly, this agent could eradicate 100% of all microorganisms in both 5 and 10 minutes and it was more effective than the other agents. 

However, it should be emphasized that the findings of the present study are not completely in agreement with the results of other studies as different brands of impression materials with different methods were being applied in these studies. One of the drawbacks of the present study is that it was an *in vitro* experimental study, which is different from clinical and *in vivo* situations. Usually impression materials remain from 3 to 5 minutes in patients' mouths, while in our study it took 60 minutes for bacteria to be attached to the samples. Also, pressure while taking an impression and saliva can alter bacterial adherence capacity. 

Since so many dentists are concerned about viruses such as HIV and HBV, further studies should be conducted to find an effective method to eradicate these kinds of pathogens.

## 5. Conclusion

According to results of the present study, it can be concluded that all three kinds of disinfectant agents effectively disinfected alginate. This disinfection capacity can be increased as time elapses except for *Pseudomonas aeruginosa* which was eradicated effectively in both 5 minutes and 10 minutes. Among different types of disinfectant agents, Epimax showed promising results in 10 minutes as it completely eradicated all kinds of microorganisms. 

## Figures and Tables

**Table 1:** Comparison of disinfectant agents and control group in 5 minutes and 1 dilution.

Disinfectants	Bacteria		
	*Candida albicans*	*Staphylococcus aureus*	*Pseudomonas aeruginosa*
	*P* value	*P* value	*P* value
Deconex-control	0.05	0.046	0.043
Hypochlorite sodium 0.525%-control	0.046	0.046	0.043
Epimax-control	0.046	0.037	0.043
Deconex-hypochlorite sodium 0.525%	0.507	0.043	0.099
Deconex-Epimax	0.507	0.034	0.099
Hypochlorite sodium 0.525%-Epimax	1.000	0.034	0.796

**Table 2:** Comparison of disinfectant agents and control agent in 10 minutes and 1 dilution.

Disinfectants	Bacteria		
	*Candida albicans*	*Staphylococcus aureus*	*Pseudomonas aeruginosa*
	*P* value	*P* value	*P* value
Deconex-control	0.046	0.050	0.034
Hypochlorite sodium 0.525%-control	0.050	0.046	0.046
Epimax-control	0.037	0.037	0.034
Deconex-hypochlorite sodium 0.525%	0.246	0.487	0.121
Deconex-Epimax	0.317	0.037	1.000
Hypochlorite sodium 0.525%-Epimax	0.121	0.034	0.121

**Table 3:** Percentage of bacterial growth prevention by different disinfectant agents in 5 and 10 minutes.

Disinfectant	Time (min)	Bacteria		
		*Candida albicans*	*Staphylococcus aureus*	*Pseudomonas aeruginosa*
Hypochlorite sodium 0.525%	5	90.62%	97.12%	99.63%
	10	96.09%	98.84%	99.54%
Epimax	5	93.74%	100%	99.52%
	10	100%	100%	100%
Deconex	5	91.40%	95.39%	99.27%
	10	99.21%	96.83%	100%
